# Digital Mental Health Resources for Asylum Seekers, Refugees, and Immigrants: Protocol for a Scoping Review

**DOI:** 10.2196/19031

**Published:** 2020-08-24

**Authors:** Buaphrao Raphiphatthana, Herdiyan Maulana, Timothy Howarth, Karen Gardner, Tricia Nagel

**Affiliations:** 1 Menzies School of Health Research Charles Darwin University Darwin Australia; 2 Faculty of Psychology State University of Jakarta Jakarta Indonesia; 3 College of Health and Human Sciences Charles Darwin University Darwin Australia; 4 University of New South Wales Sydney Australia

**Keywords:** eHealth, migrant, refugee, scoping review, immigrant

## Abstract

**Background:**

Asylum seekers, refugees, and immigrants experience a number of risk factors for mental health problems. However, in comparison to the host population, these populations are less likely to use mental health services. Digital mental health approaches have been shown to be effective in improving well-being for the general population. Thus, they may provide an effective and culturally appropriate strategy to bridge the treatment gap for these populations vulnerable to mental health risks.

**Objective:**

This paper aims to provide the background and rationale for conducting a scoping review on digital mental health resources for asylum seekers, refugees, and immigrants. It also provides an outline of the methods and analyses, which will be used to answer the following questions. What are the available digital mental health resources for asylum seekers, refugees, and immigrants? Are they effective, feasible, appropriate, and accepted by the population? What are the knowledge gaps in the field?

**Methods:**

The scoping review methodology will follow 5 phases: identifying the research question; identifying relevant studies; study selection; charting the data; and collating, summarizing, and reporting the results. Searches will be conducted in the following databases: EBSCOhost databases (CINAHL Plus with Full Text, MEDLINE with Full Text, APA PsycArticles, Psychology and Behavioral Sciences Collection, and APA PsycInfo), PubMed, and Scopus. Additionally, OpenGrey, Mednar, and Eldis will be searched for gray literature. All primary studies and gray literature in English concerning the use of information and communication technology to deliver services addressing mental health issues for asylum seekers, refugees, and immigrants will be included.

**Results:**

This scoping review will provide an overview of the available digital mental health resources for asylum seekers, refugees, and immigrants and describe the implementation outcomes of feasibility, acceptability, and appropriateness of such approaches for those populations. Potential gaps in the field will also be identified.

**Conclusions:**

As of February 2020, there were no scoping reviews, which assessed the effectiveness, feasibility, acceptability, and appropriateness of the available digital mental health resources for asylum seekers, refugees, and immigrants. This review will provide an extensive coverage on a promising and innovative intervention for such populations. It will give insight into the range of approaches, their effectiveness, and progress in their implementation. It will also provide valuable information for health practitioners, policy makers, and researchers working with the population.

**International Registered Report Identifier (IRRID):**

PRR1-10.2196/19031

## Introduction

### Background

It is well acknowledged that in comparison to host populations, asylum seekers, refugees, and immigrants are less likely to use mental health services [1-3]. Some of the reasons for their low use of mental health services are language and cultural barriers, low level of mental health literacy, concerns of stigma, experience of shame, and lack of culturally appropriate service models. Digital mental health approaches have gained much attention in the last couple of decades due to their potential cost-effectiveness, flexibility, and wide-reaching ability [4]. They may thus provide a promising strategy to bridge the treatment gap for such populations.

People have many reasons for migrating to a different country; some are volitional (immigrants), and some are forced (refugees and asylum seekers). People who voluntarily move to live away from their birth country are defined as first-generation immigrants. In the literature, second-generation immigrants can sometimes be defined as those born in the host (new) country with at least one parent born overseas or with both parents born overseas. The scoping review will adopt the latter, stricter, definition of second generations as that used in the Australian 2001 Census report [5]. An asylum seeker is a person looking for protection and resettlement in a foreign country due to fear of persecution, or because they have experienced violence or human rights violations. Those who have received protection are given refugee status [6]. The review will assess the scope of the literature on digital mental health resources for asylum seekers, refugees, and first- and second-generation immigrants living in both English and non-English–speaking countries. Henceforth, the term immigrants will be used to include both first- and second-generation immigrants. The term migrant refers to individuals who have moved away from their habitual place of residence, including movement within a country. However, for the purpose of this article, the term “migrant” will be used when referring to asylum seekers, refugees and immigrants, as a whole; it will apply only to those who have moved across international borders.

Mental health outcomes for migrants depend on multiple interacting factors, such as migration status (eg, refugee, asylum seeker, and immigrant), migration experience (pre-migration, during, and post-migration settlement factors), ethnicity or country of birth, and the host country of residency. Therefore, the prevalence of mental health outcomes for migrants is highly variable across studies [1,2]. However, there is more consistency in findings regarding the risk factors for poor mental health outcomes for migrant populations. Low socioeconomic status, poor language proficiency, experiences of intergenerational conflict, acculturation stress, racism, and perceived discrimination are some of the common factors associated with psychological distress. Particularly for refugees and asylum seekers, experiences of war, abuse, trauma, and detention centers increase the risks for developing mental health problems [3,7].

### Digital Mental Health

Digital mental health is a relatively new concept coined around the early 2000s [8]. It describes the use of information and communication technology (ICT) to deliver mental health services for health promotion/psychoeducation, prevention/early intervention, crisis intervention/suicide prevention, treatment, recovery and mutual/peer support [9]. Some examples are smartphone app-based intervention, treatment delivered via video/teleconference, online support, and telephone crisis lines. It is important to note that although the present study uses the term “digital mental health,” many other terms are used in the literature to describe the use of technology for such purposes, including e-health, e-mental health, telemedicine, and telepsychiatry. Several systematic reviews have shown the approach to be as effective as the traditional methods of care in promoting mental health and well-being in general population [10-12]. Therefore, digital mental health approaches may offer an innovative avenue to provide mental health services to a hard-to-reach population such as asylum seeker, refugee and immigrant populations.

Currently, there is no scoping review to provide an overview of the available digital mental health resources for migrant populations. One systematic review examined the efficacy of telepsychiatry for the refugee population and concluded that the intervention may be as effective as a traditional treatment [13]. Given that digital mental health resources cover a wide range of approaches (eg, smartphone apps, self-help, or clinician-guided online therapy), it would be useful to know what the available resources for migrant populations are and whether they are effective and can be successfully implemented. This information would benefit researchers to address research and treatment gaps, enhance or develop new interventions, and help health professionals and policymakers to provide culturally appropriate and effective services.

### Goal of This Study

This protocol describes the methods and processes that will be used to conduct a scoping review, which aims to provide an overview of the available digital mental health resources that are used with migrant populations worldwide. The review will aim to answer the following research questions: (1) What are the types of digital mental health resources that are available for asylum seeker, refugee, and immigrant populations? (2) Are these resources effective, feasible, appropriate, and accepted by the population? (3) What are the knowledge gaps in the field? A preliminary search for the existing scoping reviews and systematic reviews on the topic was conducted on February 12, 2020, using the following databases: Cochrane Database of Systematic Reviews, PubMed, and JBI Database of Systematic Reviews and Implementation Reports. No scoping review was found, and only 1 systematic review was found [13]. This scoping review will thus be the first to provide an overview of the available digital mental health resources for refugee and immigrant populations and the implementation outcomes of those resources (eg, feasibility, appropriateness, and acceptability).

## Methods

### Protocol Development

The protocol is developed based on the scoping review framework proposed by Arksey and O’Malley [14] and the enhancements proposed by Peters et al [15]. It involves the following 5-stage process: (1) identify and align the study’s objective and research questions; (2) develop and align the inclusion criteria with the objectives and research questions to help identify relevant studies; (3) develop a systematic approach to evidence searching, selection, extraction, and charting; (4) chart the data from selected studies; and (5) summarize the evidence in relation to the research objective and questions. The recommendations of the PRISMA-P (Preferred Reporting Items for Systematic Reviews and Meta-Analyses Protocols) are provided in Multimedia Appendix 1. We have not registered the document with PROSPERO, as scoping reviews are currently ineligible for registration in the database.

### Inclusion Criteria

#### Type of Studies

The term “e-health” was first used at the 7th International Congress on Telemedicine and Telecare in London, at the end of November 1999 [8]. Telemedicine, one of the first aspects of ehealth was first entered into the Medical Subject Heading (MeSH) list of the National Library of Medicine in 1990. Therefore, we will include all primary studies published in English from January 1, 1990, to December 31, 2019. We will aim to include all studies conducted worldwide. However, we are limited to those published in English, as it is the only common language across the authors of this paper. All research designs will be included, although opinion pieces and reviews will only be used for reference searching and will not be included in outcome analyses. If full studies are not available, they will be requested from the corresponding author. When a full report is unobtainable, we will include published abstracts if there is sufficient information to assess study eligibility. Gray literature will also be included.

#### Participants

Studies that report on migrant populations (ie, asylum seekers who live outside their country of citizenship, refugees, and first- and second-generation immigrants) will be included. As previously mentioned, second-generation immigrants are those with both parents born overseas. Thus, the included studies will be those reporting on samples that meet one of the following criteria: (1) born in a country different from their residential country and (2) have both parents born in a country different from the person’s residential country. Populations that are not considered as refugees or immigrants such as internal migrants, host populations, and indigenous populations will be excluded.

#### Intervention

All types of digital mental health approaches that use ICT to deliver services targeting mental health issues for migrant populations will be included. These include the following interventions: internet-based interventions, gamification-based interventions, email, videoconferencing, telephone interventions, mobile app-based interventions, virtual reality, and any other digital devices used to address mental health. The devices can be used for delivering services of mental health promotion/psychoeducation, prevention/early intervention, crisis intervention/suicide prevention, treatment, recovery, and mutual/peer support. Technologies used to assess an individual’s mental health outcomes as part of service delivery will be included. However, assessments carried out via digital technology for the purpose of assessing population-based mental health outcomes (eg, establishing prevalence of mental health illness in a population) will be excluded. Additionally, technology used for sending appointment reminders and communication of test results will also be excluded.

#### Comparators

Studies may or may not include comparison of processes or outcomes against other types of interventions, traditional methods of mental health services, or matched control groups. Given that comparators and control groups may vary significantly across the selected studies, we will note whether comparator groups are included and will describe them in detail.

#### Outcomes

We will include all outcome measures of mental health/social and emotional well-being to examine the effectiveness of digital mental health approaches in promoting migrant populations’ mental health. The language and validity of the measures used with the population will also be noted. The implementation outcomes, specifically those of feasibility, appropriateness, and acceptability for the population will be included. Outcomes not related to mental health/social and emotional well-being (ie, medical/physical health outcomes) will be excluded.

### Search Methods for Identification of Studies

#### Search Strategy

A 3-step search strategy, as recommended by the Joanna Briggs Institute [16], will be used. First, at least 2 independent reviewers will conduct an initial limited search of the 3 databases relevant to the topic (EBSCOhost databases, PubMed and Scopus). Text words contained in the title, abstract, and index term of the retrieved articles will be assessed for additional relevant keywords. The reviewers will then discuss and finalize the keywords to be used for the second search, which will be conducted by at least 2 reviewers independently, across the following databases: EBSCOhost databases (CINAHL Plus with Full Text, MEDLINE with Full Text, APA PsycArticles, Psychology and Behavioral Sciences Collection, APA PsycInfo); PubMed; and Scopus. The key words will also be searched in OpenGrey, Mednar, and Eldis for gray literature. Lastly, the reference list from relevant articles chosen for potential inclusion, opinion pieces, and review studies will be handsearched to identify further relevant studies. The corresponding authors will be contacted for the full article or further information, if required. The search strategies for the 3 databases: EBSCOhost databases, Scopus, and PubMed are presented in Multimedia Appendix 2.

#### Study Selection

At least 2 reviewers will independently apply the inclusion and exclusion criteria to the title and abstract of the studies retrieved through the search strategy. The inclusion criterion is the use of ICT to deliver services targeting mental health problems for migrant populations. The services provided can be for mental health promotion/psychoeducation, prevention/early intervention (including an individual’s mental health assessment), crisis intervention/suicide prevention, treatment, recovery, and mutual/peer support. Exclusion criteria are as follows: population not from refugee and immigrant backgrounds; interventions not related to mental health and well-being (ie, those for physical health); and ICT used for population-based mental health assessment and for sending notifications purposes.

The full text of the articles meeting the inclusion criteria at the title and abstract level will then be retrieved, read, and assessed by at least 2 independent reviewers. The following data will be extracted: demographic information of the population; the type of the digital mental health approach; the measures used to assess mental health and social and emotional well-being; the measures’ outcomes; and the digital mental health approach’s outcomes with respect to its feasibility, appropriateness, and acceptability. The studies will also be assessed for exclusion criteria as described above. If there is any disagreement, a third reviewer will be asked to review the article. Corresponding authors of the studies will be contacted if further clarifications are needed. Decisions and reasons for exclusions will be documented. Endnote X9 (Clarivate Analytics) will be used to manage references throughout the process.

### Data Extraction

At least 2 independent reviewers will use a standardized data collection form to collect relevant data (Textbox 1) from each study. Corresponding authors will be contacted for missing data.

**Data to be extracted from selected studies.**

- Study citation details: authors, date, title, journal, volume, issue, and pages.
- Country of origin (where the study was conducted).
- Study’s aims/objective/purpose.
- Study’s design.
- Specific details of the study’s sample if available (age; sex; ethnicity; country of birth; year of arriving in the host country; first- or second-generation immigration; languages spoken; immigration status, eg, refugee, asylum seeker; and sample size).
- The type of digital mental health intervention (eg, smartphone app, online support, online therapy, or website).
- The type of mental health services: mental health promotion/psychoeducation, prevention/early intervention, crisis intervention/suicide prevention, treatment, recovery, and mutual/peer support.
- Mode of delivery: self-guided, clinician supported, or both.
- Mental health and well-being outcomes (effectiveness of the intervention) and details of how they were measured (ie, quantitatively or qualitatively, which language was used, whether a translator was used, and whether the measurement method has been validated for that specific population).
- Outcomes of the intervention’s feasibility, acceptability, and appropriateness for the population.

### Charting the Data

Simple descriptive tables will be used to report basic information of the selected studies (ie, citation, objectives, the sample’s demographic information, and relevant findings/outcomes). Thematic analysis as outlined by Braun and Clarke [17] will be used to identify categories and central themes from the literature review. No complex statistical analyses such as meta-analyses will be performed. This is because the review aims to be inclusive and will broadly describe the literature, and intervention outcomes may be too heterogeneous for such rigorous analyses. Reported outcomes will be extracted, analyzed separately, and compared across migrant groups (asylum seekers, refugees, first- and second-generation immigrants).

## Results

The scoping review will provide an overview of the digital mental health resources available for migrant populations and will describe implementation outcomes with respect to their feasibility, acceptability, and appropriateness for different populations. Therefore, data on the breadth of digital mental health interventions addressing migrant populations and their effectiveness, feasibility, appropriateness, and acceptability will be extracted, analyzed, and presented according to Arksey and O’Malley’s [14] methods of reporting. A narrative thematic summary of the data will also be presented, and gaps in the literature will be highlighted.

## Discussion

This scoping review will provide a comprehensive overview of the available digital mental health resources for migrant populations and summarize the outcomes in this field in terms of their effectiveness, feasibility, acceptability, and appropriateness for different migrant populations. This information will benefit policymakers, researchers, and service providers working with people from migrant backgrounds. It can be used to inform future policies and strategies for bridging the treatment gap for this population vulnerable to mental health risks. It will also provide evidence for potentially effective and culturally appropriate approaches for service providers to use or recommend. Moreover, since this scoping review is first of its kind in this research field, it will highlight important gaps in the literature, providing guidance and directions for future research.

## Appendix

Multimedia Appendix 1 PRISMA-P checklist for systematic reviews.

Multimedia Appendix 2 Study search strategy.

## Abbreviations

ICT: information and communication technology
MeSH: Medical Subject Heading
PRISMA-P: Preferred Reporting Items for Systematic Reviews and Meta-Analyses Protocols
