# Supplementary material for: Digital Mental Health Resources for Asylum Seekers, Refugees, and Immigrants: Protocol for a Scoping Review
Source: JMIR Res Protoc. 2020 Aug 24;9(8):e19031. doi: 10.2196/19031 (PMC7477666; doi:10.2196/19031)
Supplement: Multimedia Appendix 2 [file resprot_v9i8e19031_app2.pdf]

Supplementary File 2: Search Strategy

EBSCOhost databases:

Selected databases: CINAHL Plus with Full text, MEDLINE with full text, APA PsychArticles, Psychology and Behavioural sciences collection, APA PsychInfo.

Published date: Jan 1990 – December 2019

Language: English

Resource types: All results

Search terms:

"e-Mental Health" OR e-Health OR "digital mental health" OR telemedic* OR telehealth OR "telemental health" OR mhealth OR mtherapy OR "online therapy" OR "online intervention" OR e-therapy OR "internet intervention" OR "computer-assisted therapy" OR "online self-help" OR iCBT OR "website intervention" OR "web-based treatment"

AND

CALD OR "culturally and linguistically diverse" OR migrant* OR immigrant* OR emigrant* OR refugee* OR "asylum seeker" OR multiling* OR biling* OR cross-cultural* OR multi-cultural* OR displace* OR Foreign*

AND

Mental health OR well-being OR distress OR mental disorder* OR mental illness* OR psych*

Scopus:

(TITLE-ABS-KEY ("e-Mental Health") OR TITLE-ABS-KEY (e-health) OR TITLE-ABS-KEY ("digital mental health") OR TITLE-ABS-KEY (telemedic*) OR TITLE-ABS-KEY (telehealth) OR TITLE-ABS-KEY ("telemental health") OR TITLE-ABS-KEY (mhealth) OR TITLE-ABS-KEY (mtherapy) OR TITLE-ABS-KEY ("online therapy") OR TITLE-ABS-KEY ("online intervention") OR TITLE-ABS-KEY (e-therapy) OR TITLE-ABS-KEY ("internet intervention") OR TITLE-ABS-KEY ("computer-assisted therapy") OR TITLE-ABS-KEY ("online self-help") OR TITLE-ABS-KEY (icbt) OR TITLE-ABS-KEY ("website intervention") OR TITLE-ABS-KEY ("web-based treatment")) AND (TITLE-ABS-KEY (cald) OR TITLE-ABS-KEY ("culturally and linguistically diverse") OR TITLE-ABS-KEY (migrant*) OR TITLE-ABS-KEY (immigrant*) OR TITLE-ABS-KEY (emigrant*) OR TITLE-ABS-KEY (refugee*) OR TITLE-ABS-KEY ("asylum seeker") OR TITLE-ABS-KEY (multiling*) OR TITLE-ABS-KEY (billing*) OR TITLE-ABS-KEY (cross-cultural*) OR TITLE-ABS-KEY (multi-cultural*) OR TITLE-ABS-KEY (displace*) OR TITLE-ABS-KEY (foreign*)) AND (TITLE-ABS-KEY ("mental health") OR TITLE-ABS-KEY (well-being) OR TITLE-ABS-KEY (distress) OR TITLE-ABS-KEY (mental AND disorder*) OR TITLE-ABS-KEY (mental AND illness*) OR TITLE-ABS-KEY (psych*)) AND PUBYEAR > 1989 AND PUBYEAR < 2020 AND (LIMIT-TO (LANGUAGE , "English"))

Supplementary File 2: Search Strategy

PubMed:

(("mobile applications"[MeSH Terms] OR ("mobile"[All Fields] AND "applications"[All Fields]) OR "mobile applications"[All Fields]) OR ("telemedicine"[MeSH Terms] OR "telemedicine"[All Fields]) OR ("therapy, computer-assisted"[MeSH Terms] OR ("therapy"[All Fields] AND "computer-assisted"[All Fields]) OR "computer-assisted therapy"[All Fields] OR ("therapy"[All Fields] AND "computer"[All Fields] AND "assisted"[All Fields]) OR "therapy, computer assisted"[All Fields]) OR ("videoconferencing"[MeSH Terms] OR "videoconferencing"[All Fields])) AND (("refugees"[MeSH Terms] OR "refugees"[All Fields]) OR ("emigrants and immigrants"[MeSH Terms] OR ("emigrants"[All Fields] AND "immigrants"[All Fields]) OR "emigrants and immigrants"[All Fields]) OR ("transients and migrants"[MeSH Terms] OR ("transients"[All Fields] AND "migrants"[All Fields]) OR "transients and migrants"[All Fields]) OR "culturally and linguistically diverse"[All Fields] OR "CALD"[All Fields] OR ("refugees"[MeSH Terms] OR "refugees"[All Fields] OR ("asylum"[All Fields] AND "seeker"[All Fields]) OR "asylum seeker"[All Fields]) OR ("foreign professional personnel"[MeSH Terms] OR ("foreign"[All Fields] AND "professional"[All Fields] AND "personnel"[All Fields]) OR "foreign professional personnel"[All Fields]) OR "multilingual*"[All Fields] OR "Cross cultural"[All Fields] OR (displace[All Fields] OR displaced[All Fields] OR displacedaway[All Fields] OR displacement[All Fields] OR displacements[All Fields]) OR (foreign[All Fields] OR foreignborn[All Fields] OR foreigner[All Fields] OR foreignlanguagepatients[All Fields])) AND (("mental disorders"[MeSH Terms] OR ("mental"[All Fields] AND "disorders"[All Fields]) OR "mental disorders"[All Fields]) OR ("mental health"[MeSH Terms] OR ("mental"[All Fields] AND "health"[All Fields]) OR "mental health"[All Fields]) OR ("psychotherapy"[MeSH Terms] OR "psychotherapy"[All Fields]) OR ("psychophysiology"[MeSH Terms] OR "psychophysiology"[All Fields]) OR ("psychiatry"[MeSH Terms] OR "psychiatry"[All Fields]))
